# Mass cytometry dissects T cell heterogeneity in the immune tumor microenvironment of common dysproteinemias at diagnosis and after first line therapies

**DOI:** 10.1038/s41408-019-0234-4

**Published:** 2019-08-28

**Authors:** Taxiarchis V. Kourelis, Jose C. Villasboas, Erik Jessen, Surendra Dasari, Angela Dispenzieri, Dragan Jevremovic, Shaji Kumar

**Affiliations:** 1 Division of Hematology, Department of Medicine, Mayo Clinic, Rochester, MN USA; 2 Department of Health Sciences Research, Mayo Clinic, Rochester, MN USA; 3 Department of Laboratory Medicine and Pathology, Mayo Clinic, Rochester, MN USA

**Keywords:** Translational research, Cancer microenvironment

## Abstract

Dysproteinemias progress through a series of clonal evolution events in the tumor cell along with the development of a progressively more “permissive” immune tumor microenvironment (iTME). Novel multiparametric cytometry approaches, such as cytometry by time-of-flight (CyTOF) combined with novel gating algorithms can rapidly characterize previously unknown phenotypes in the iTME of tumors and better capture its heterogeneity. Here, we used a 33-marker CyTOF panel to characterize the iTME of dysproteinemia patients (MGUS, multiple myeloma—MM, smoldering MM, and AL amyloidosis) at diagnosis and after standard of care first line therapies (triplet induction chemotherapy and autologous stem cell transplant—ASCT). We identify novel subsets, some of which are unique to the iTME and absent from matched peripheral blood samples, with potential roles in tumor immunosurveillance as well as tumor immune escape. We find that AL amyloidosis has a distinct iTME compared to other dysproteinemias with higher myeloid and “innate-like” T cell subset infiltration. We show that T cell immune senescence might be implicated in disease pathogenesis in patients with trisomies. Finally, we demonstrate that the early post-ASCT period is associated with an increase of senescent and exhausted subsets, which might have implications for the rational selection of post-ASCT therapies.

## Introduction

Multiple myeloma (MM) and its precursors, monoclonal gammopathy of undetermined significance (MGUS) and smoldering MM (SMM), progress through a series of clonal evolution events in the tumor cell and the development of a progressively more “permissive” tumor microenvironment (TME)^1^. The term TME is broad and encompasses immune and non-immune cellular components. The immune TME (iTME) in MM consists of a complex network of immune cells that interact with the malignant clone and with each other to sometimes promote and other times inhibit malignant plasma cell growth. Prior studies have demonstrated the role of T cell and innate immune populations in MM pathogenesis^2^. Among these, immune profiling studies using cytometry-based assays represent powerful exploratory tools to identify potential immune subsets of interest that correlate with clinical outcomes^3^ and could be targeted with therapy. For instance, T cell dysfunction has been implicated in SMM progression^4^ and may be reversed to some extent by lenalidomide. Myeloid populations are implicated in osteoclastogenesis and MM progression^5^.

Novel, multiparametric cytometry approaches, such as cytometry by time-of-flight (CyTOF) or high parameter flow cytometry can simultaneously asses more than 40 proteins at a single-cell level. Combined with novel, machine-learning-based gating algorithms, this approach can rapidly characterize previously unknown phenotypes in the iTME of tumors in a data-driven way free of the biases introduced by manual gating.

In this study, using CyTOF we evaluated the iTME, of newly diagnosed MM (NDMM), MGUS, SMM, and light chain (AL) amyloidosis at diagnosis and after standard first line therapies.

## Materials and methods

### Patients and samples

After institutional IRB approval, we included 81 unique dysproteinemia patients with 108 corresponding bone marrow (BM) samples at diagnosis and after therapy (supplemental Fig. 1). Of 14 NDMM patients, 12 and 10 patients had matched samples post-induction and at 100 days post-autologous stem cell transplant (ASCT), respectively. We also included 12 healthy donors (HD). HD and dysproteinemia patients were not age-matched and for this reason we only included the HD group in a limited number of analyses. We considered patients with AL amyloidosis and >10% BMPCs separately given their different natural history^6^. Thirty-five peripheral blood (PB) samples corresponding to the same patients at various stages (diagnosis, post-therapy) were also used (supplemental Fig. 1) along with 7 PB HD, for a total of 150 unique samples.

### Antibody panel

Our antibody panel included 33 antibodies directed against well-characterized surface markers (supplemental Fig. 2). Mass cytometry antibody–metal conjugate combinations are detailed in supplemental Table 1. Sample preparation, antibody staining, and CyTOF acquisition are detailed in the supplemental methods.

### Mass cytometry data analysis

Initial data processing and quality control were performed by us, as well as by well informatics experts at Cytobank. We defined a bead singlet population as the reference population for normalization in all the samples^7^. Files were normalized using the premessa R package. Before normalization, we observed a batch effect in the dataset by plotting the median expression of the gated bead singlet population in the 140 bead channel. A one-way analysis of variance (ANOVA) test showed that the batch variable was significantly correlated (*p* < 2 × 10^−16^) with the median expression of the bead singlet population. After normalization, we observed the coefficient of variation of the median expression across samples decreased from 11.75% to 6%. Normalized files were then uploaded into Cytobank and data were gated to exclude normalization beads and to include live, single cells (supplemental Fig. 3). Our median CD3/CD19 doublet rate after this gating strategy was 0.1% (range 0.008–0.9%). We then further removed doublets by removing CD3/CD19 and CD3/CD14 double positive cells. Only CD45+ immune cells were considered for downstream analyses. Malignant plasma cells are commonly CD45− but CD45+ malignant cells were clustered separately by the gating algorithms based on their CD138, CD38, and CD56 expressions.

Signal intensities for each mass channel were arcsinh transformed with a cofactor of 5. Clustering analyses were performed in Cytobank using FlowSOM^8^. We employed the following methods to ensure cluster stability: (a) FlowSOM clustering was repeated five times for each parent population using a different seed every time, (b) FlowSOM identified metaclusters were visualized using viSNE^9^, which also served as a validation method, since viSNE uses a conceptually different machine-learning algorithm to that of FlowSOM: if a FlowSOM identified (meta)cluster did not form a discrete viSNE island then it was not considered, and (c) we dichotomized the dataset into a discovery and a validation cohort and only clusters identified in both were considered.

Separate FlowSOM analyses were performed on all samples on the following manually gated populations: (a) all CD45+, live, single cells, and to better dissect T cell heterogeneity, (b) CD45/CD3/CD8+, live, single cells, and (c) CD45/CD3/CD4+ live, single cells. We used the lowest number of cells available in a sample and included the same number of cells/sample when clustering to ensure equal representation of cells across samples. For BM CD45+ cells we included all samples (12,822 cells/sample) and all markers were used for clustering except: CD45. For BM CD8+ and CD4+ T cells, 1355 and 2076 cells/sample were clustered, respectively (one sample was excluded from CD8+ analyses due to low event counts) and all markers except the following were included: CD45, CD3, CD8, CD4, CD11c, CD123, CD138, CD14, CD163, CD19, PDL-1. For visualization with viSNE 2000 events/sample and 1355 cells/sample were used for CD8+ and CD4+ T cells, respectively. The same clustering and visualization parameters were used to cluster PB samples except for including a higher number of cells for each population upon initial clustering as follows: CD45+ 28,896 events/sample, CD8+ 5079 events/sample, and CD4+ 4067 cells/sample. For visualization 2000 events/sample were included. All cytometry data can be made available upon request.

### Statistical analyses

Statistical analyses were performed using JMP (SAS) v 14. To compare continuous and categorical variables the Wilcoxon rank-sum and Fischer’s exact tests were used, respectively. Correlations between immune cell frequencies across patients were assessed using the Pearson correlation coefficient. Hierarchical clustering and principal component analyses (PCA) were performed using R (v.3.3.2). Time to event analyses were performed using the Kaplan–Meier method. Given the multiple comparisons performed, *p* values of < 0.001 were considered statistically significant whereas *p* values of < 0.05 were considered indicative of potential trends.

## Results

The baseline clinical and laboratory characteristics of patients are shown in the Table 1. HD included 5 male and 7 female patients with a median age of 29 (range 20–34).

**Table 1:** Baseline characteristics of patients

Characteristic, *N* = 66	*N* (%)
Male sex	43 (65%)
Age, median (range)	66 (28–89)
Heavy chain restriction	
IgA	10 (15%)
IgM	2 (3%)
IgG	31 (47%)
Light chain only	23 (35%)
Light chain restriction	
Kappa light chain	33 (50%)
Lambda light chain	33 (50%)
% Bone marrow plasma cells, median (range)	12.5 (0–90)
FISH^a^	
High risk FISH^b^	17 (31%)
Trisomies^c^	15 (28%)
t (11;14)^d^	18 (33%)
Myeloma, receiving induction therapy	14 (100%)
Cycles of induction	4 (2–5)
Lenalidomide, bortezomib, dexamethasone	13/14
Pembrolizumab, lenalidomide, dexamethasone	1/14
Amyloidosis, receiving induction therapy	7/28
Cycles of induction	4 (2–7)
Lenalidomide, bortezomib, dexamethasone	1/7
Lenalidomide, dexamethasone	1/7
Cyclophosphamide, bortezomib, dexamethasone	5/7

^a^Of 54 with available data

^b^−17p, t(4;14), +1q, t(14;16), t(14;20)

^c^In which seven trisomies were the sole abnormality

^d^In which 15 t(11;14) was the sole abnormality

### Mass cytometry reveals a diverse immune microenvironment in dysproteinemias with phenotypes associated with tumor tolerance and immunosurveillance

**Fig. 1:**
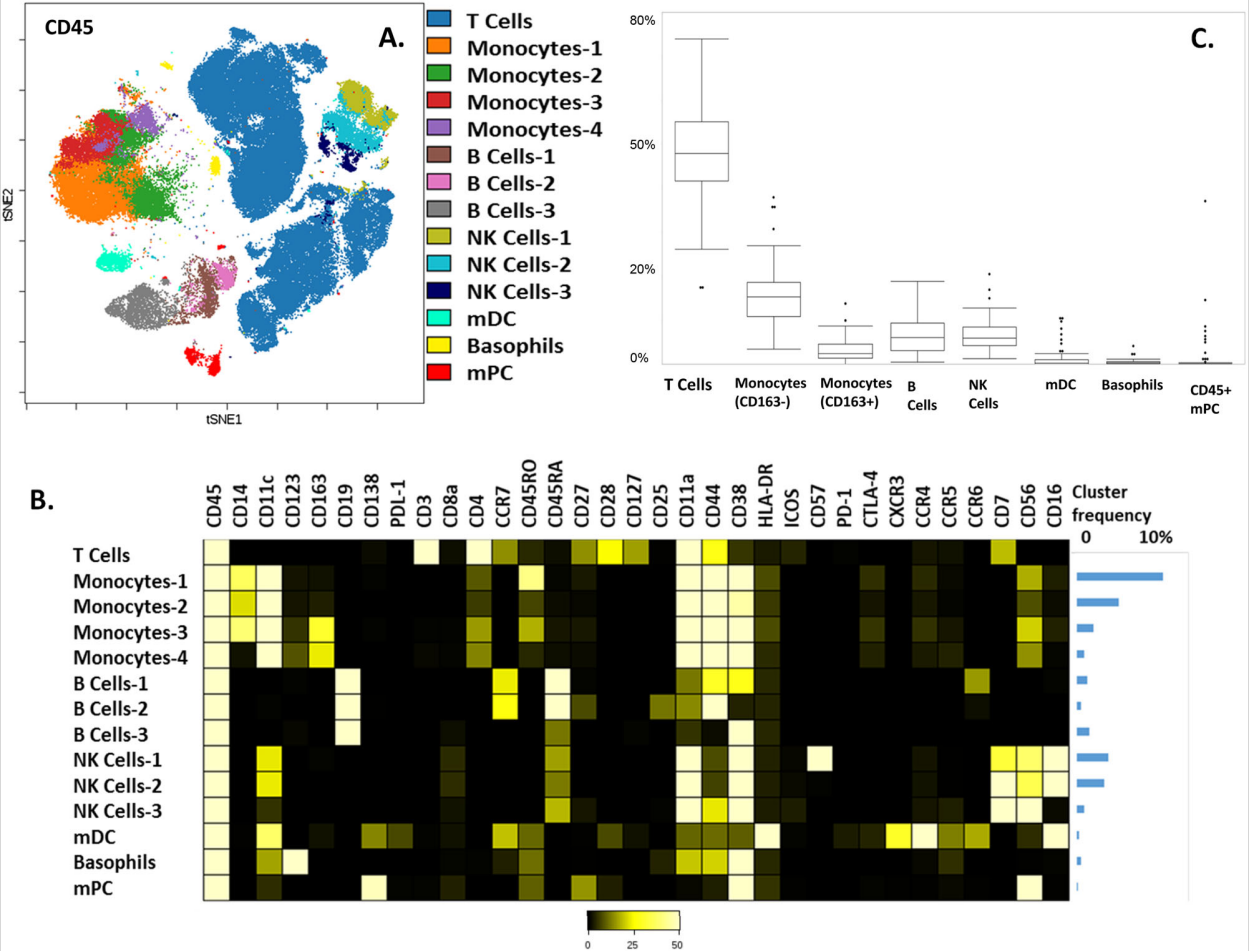
Characterizing the main immune components of the marrow microenvironment in common dysproteinemias. **a** viSNE map of 132,000 CD45+ cells (2000 cells/patient) from 66 patients with newly diagnosed dysproteinemias. Lineage negative populations are not shown for clarity. **b** Heatmap showing expression of markers for each cluster; relative median frequencies of total CD45+ cells are shown as a bar graph on the right (T cell frequency not shown). **c** Box plots showing the frequency of each major immune cell subset. mDC myeloid dendritic cells, NK natural killer cells, mPC malignant plasma cells

FlowSOM identified 12 distinct immune clusters in the CD45+ compartment (Fig. 1). T cells were the most abundant immune cell population in the iTME of dysproteinemias. Detailed frequencies of immune subsets and contour plots of main lineage markers are shown in supplemental Table 2 and supplemental Fig. 4, respectively. Four monocyte populations were identified based on the levels of CD45RO, CD163, and CD14 expression: monocytes-1 (CD45RO high), monocytes-2 (CD45RO low), monocytes-3 (CD14/CD163+), and monocytes-4 (CD14low/CD163+). CD45RO high monocytes were the dominant population and are thought to represent an activated population transmigrating to peripheral tissues^10^ that increase with age^11^. Of the two, M2 polarized, CD163+ monocyte clusters, the CD14low subset is thought to represent a maturing population^12^. Three B cell populations were identified: B cells-1 (CCR6 positive), B Cells-2 (CD25/CD27 positive), and B Cells-3 (CD38 high). B Cells-1 are thought to represent memory B cell precursors with low affinity for antigens^13^. B Cells-2, the least abundant B cell subset, are highly activated, Ig class switched memory (CD27+) B cells with the capacity to produce higher levels of IL-10^14^, a cytokine associated with increased malignant plasma cell proliferation^15^ and worse prognosis in MM^16^. B cells-3, the most abundant B cell subset, consisted of transitional B cells. Finally, three natural killer (NK) cell populations were identified. NK cell-1 and NK cell-2 were the most abundant and were both CD16-positive populations differentiated by their CD57 expression (high in NK-Cells-1). CD57+ NK cells are thought to represent a mature subset with higher cytotoxic capacity, greater responsiveness to signaling via CD16 and associated with improved outcomes in several solid and hematologic malignancies^17^. NK Cells-3, a CD56 high, CD16 negative population, represent weakly cytolytic, cytokine producing NK cells that are thought to have immuno-regulatory properties^18^.

**Fig. 2:**
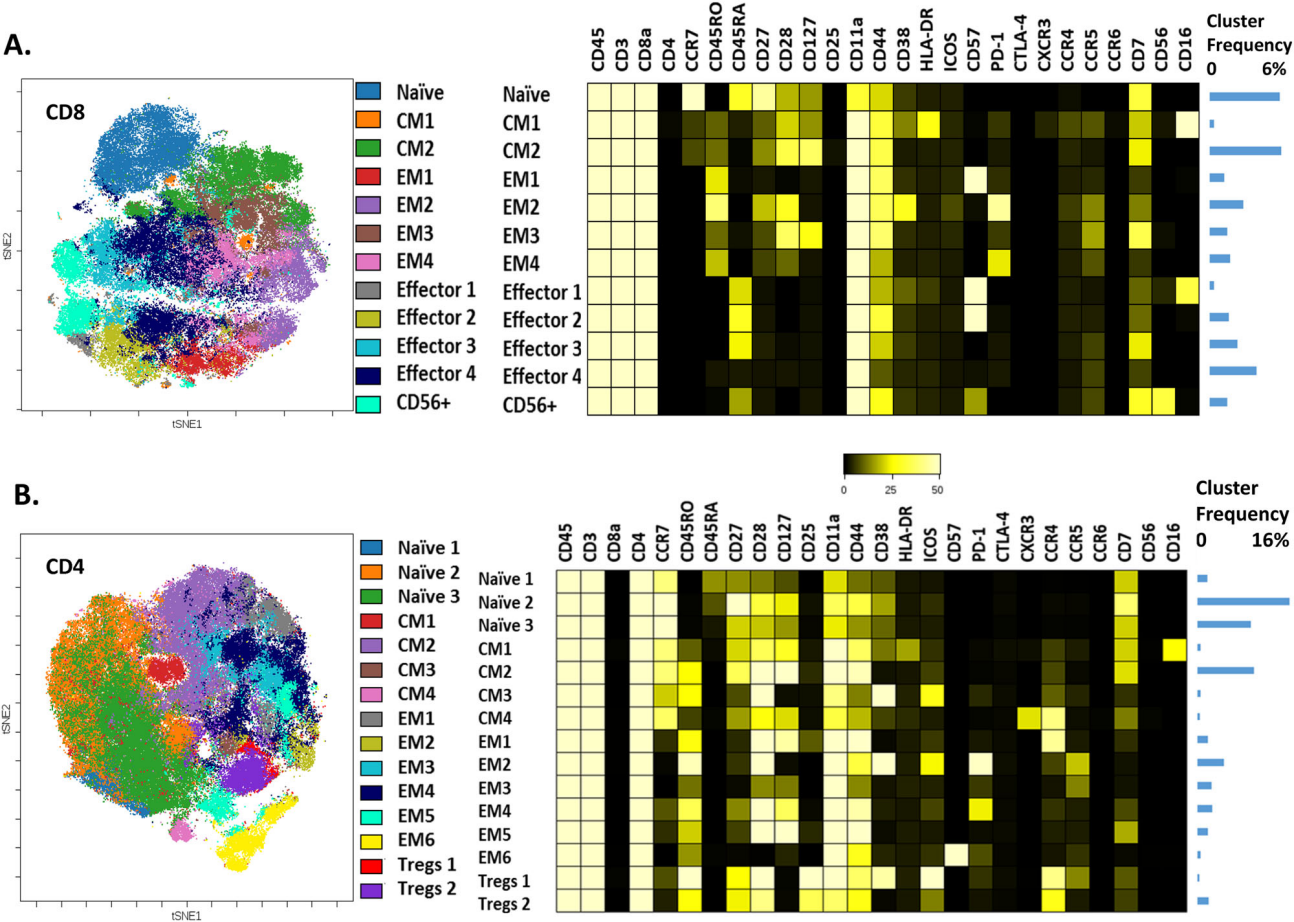
CyTOF analyses of T cells in the tumor microenvironment of common dysproteinemias. **a** viSNE map and respective heatmap of 89,430 (1355 cells/patient) CD8+ T cells from 66 patients with newly diagnosed dysproteinemias. **b** viSNE map and respective heatmap of 132,000 (2000 cells/ patient) CD4+ T cells from 66 patients with newly diagnosed dysproteinemias. Markers not expressed on T cells are not shown for clarity. Relative median frequencies of total CD3+ cells are shown as a bar graph on the right. CM central memory, EM effector memory, T regs T regulatory cells

Separate FlowSOM clustering was performed on manually gated CD8 and CD4 T cell subsets to better dissect their heterogeneity. When considering CD8+ and CD4+ T Cells, 12 and 15 distinct clusters were identified, respectively (Fig. 2). We observed several similar phenotypes between CD8 and CD4 cells. The central memory (CM)-1 CD4 and CD8 populations, were both characterized by HLA-DR and CD16 co-expression. These subsets, along with the effector 1 CD8 population, that had a phenotype consistent with terminal differentiation (CD57+, CD27/CD28−), were the only ones characterized by CD16 positivity and are thought to represent “innate-like” subsets^19,20^ that can be activated in the absence of T cell receptor stimulation.

Other subsets with similar phenotypes included the terminally differentiated (CD57+) effector memory EM1 in CD8+ and EM6 subsets in CD4+ T cells. Exhausted (PD1+) subsets with common phenotypes included the EM2 subset (common in CD4 and CD8), which was characterized by CD38 co-expression. Maximal T cell recruitment and T cell-mediated antitumor responses rely upon CCR5 expression in both CD4+ and CD8+ T cells^21,22^. We identified several CCR5-expressing subsets (CD8:EM2, EM3, EM4; CD4:EM2, EM3), some of which were PD1+.

Other subsets of interest included the CXCR3/CCR4+ CM4 CD4 subset with a Th1-like phenotype and the ICOS/CD38+ positive Treg-1 subset. The former is thought to have an immunosurveilling role in MM^23^. The latter is thought to be a highly immunosuppressive T regulatory (Treg) subset associated with poor outcomes in several malignancies^24–27^.

### Several immune T cell subsets in the iTME are distinct from those in the PB

In the PB, several CD45+ phenotypes were common to those in the BM (supplemental Fig. 5) except for some activated phenotypes (HLA-DR+ monocyte and B cell subsets) as expected. CD38+ plasmacytoid dendritic cells (CD123/HLA-DR+/CD11c−) were detectable only in the PB and basophils (CD123/CD11c+/HLA-DR−) only in the BM. The CD8 CM1 subset was not detectable in the PB. PD-1 and CD38 co-expressing T cell subsets (PB EM2 CD8 and CD4 subsets) were again noted. However, the ICOS-positive Tregs in the PB were CD38 negative. In addition, CCR5+, exhausted (PD-1+) T cell subsets were not detectable in the periphery, suggesting that T cell exhaustion of tumor surveilling populations and Treg activation through CD38 might be immune suppressive mechanisms exclusive to the iTME (supplemental Fig. 6).

### Myeloid and innate-like CD4 T cell subsets are more abundant in the iTME of AL amyloidosis

**Fig. 3:**
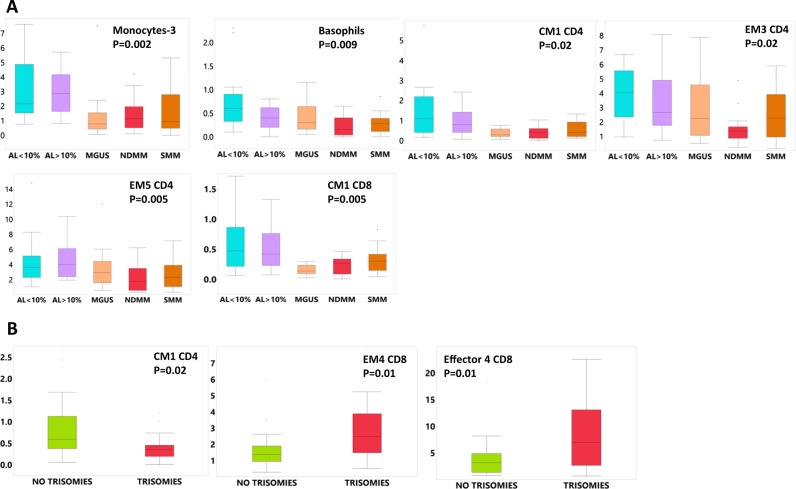
Boxplots showing differential abundance (% of CD45+ cells, T cell subset frequencies are % of total CD3 cells) of differentially represented immune subsets (across group *p*-value is shown, significant differences mentioned in the text). **a** At diagnosis and **b** according to the presence/absence of trisomies. AL >or <10% light chain amyloidosis with > or<10% bone marrow plasma cells, respectively, CM central memory, EM effector memory, MGUS monoclonal gammopathy of undetermined significance, NDMM newly diagnosed multiple myeloma, SMM smoldering multiple myeloma

We then considered differential abundance of immune subsets at diagnosis across groups and identified interesting trends. M2 polarized monocytes (monocytes-3) were increased in high (≥10%) and low (<10%) plasma cell burden AL amyloidosis compared to all other dysproteinemias (Fig. 3a). Amyloid-clearing^28^, CD163+ macrophage infiltration has been reported in other tissues^28–32^ of patients with amyloidosis and could account for this observation. The CM1 CD8 and CD4 subsets were also increased compared to myeloma and MGUS. Furthermore, basophils, the CCR5+ EM3 CD4 and CD28+ EM5 CD4 subsets were increased in AL amyloidosis compared to MM. Finally, we noted no differences when we considered groups according to plasma cell burden: ≥10%: AL amyloidosis, SMM, NDMM and <10%: AL amyloidosis, MGUS (data not shown).

### The iTME of patients with trisomies is populated by more senescent/terminally differentiated and exhausted T cell subsets

We then evaluated whether cytogenetic features of the malignant plasma cells correlate with specific changes in the iTME (Fig. 3b). We considered three groups: patients with high risk genetic abnormalities, patients with t (11;14) and patients with trisomies. We found that only in patients with trisomies the CM1 CD4 population was decreased and the PD-1+ and terminally differentiated EM4 and Effector 4 populations, respectively, were increased, although these differences did not reach our predetermined significance cut-off. Since trisomies are associated with higher rates of progression to MM when seen in MGUS/SMM we then considered differences between patients with SMM or MGUS that had an increase in their monoclonal protein of at least 25% within 2 years from diagnosis versus those who did not. We found no differences between these groups. In the non-progressing group, we evaluated one patient in more detail. He had presented with high risk monoclonal kappa smoldering myeloma (kappa serum free light chain (sFLC) level of 957 mg/dL) but elected against therapy. During his most recent follow-up, 6 years from diagnosis, he has demonstrated ongoing “spontaneous” hematologic response without any therapy (most recent kappa free light chain level of 212 mg/dL). This patient’s levels of several terminally differentiated T cell subsets (CD4: EM6, CD8: EM1, Effector 1,2,3 and CD56+ T cells) and CD163+ monocyte populations were among the lowest in the group. He also had the highest levels of the CM1 CD4 population (supplemental Fig. 7).

### Exhausted and senescent/terminally differentiated subsets are increased in the iTME of dysproteinemias early after ASCT

After lenalidomide/bortezomib-based induction therapy the monocyte-1 and all CD163+ monocyte populations (monocytes 3 and 4) increased, and total NK cells decreased in patients with MM (*p* < 0.05) (supplemental Fig. 8). Two CCR5+ T cell populations (EM3 CD4 and EM3 CD8) increased.

**Fig. 4:**
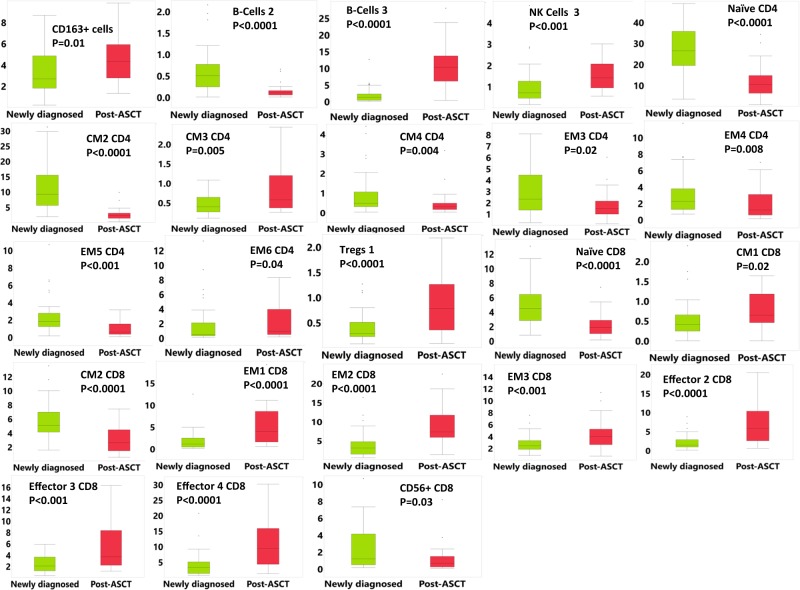
Boxplots showing differential abundance of immune subsets at diagnosis and after autologous stem cell transplant in patients with newly diagnosed multiple myeloma and AL amyloidosis. T cell subset frequencies are percent of total CD3 cells. Only significantly different subsets are shown for clarity. ASCT autologous stem cell transplant, CM central memory, EM effector memory, NK natural killer

Of 29 patients who received ASCT, most achieved deep clonal remissions: 22 achieved a complete remission (3 had MRD-negative disease), 5 achieved a very good partial remission, and 2 a partial remission. Except for total CD163+ monocyte populations, that did not increase further post-ASCT in AL amyloidosis, all other differences noted were common between AL and MM and most reached statistical significance. Therefore, we considered them together for these analyses. Post-ASCT, CD163+ monocytes, the transitional B-cell-3 subset and the CD16− NK-cell 3 subset increased whereas the B-cell 2 subset decreased (Fig. 4).

T cell populations that decreased included the CD28+ CM2 CD4, EM5 CD4, CM2 CD8 populations and the Th1-like CM4 CD4 population. Terminally differentiated populations that increased included the EM6 CD4, EM1 CD8 and effector 2, 3, and 4 CD8 populations. The exhausted EM2 CD8 and the ICOS+ Tregs-1 population also increased post-ASCT. However, the activated CM3 CD4 subset increased and the exhausted EM4 CD4 and terminally differentiated CCR5+ EM3 population decreased (Fig. 4).

We finally considered patients who relapsed within 12 months from their ASCT, a group thought to have inferior outcomes and associated with more aggressive disease biology^33^. In early progressors the CM4 CD4 population (Th1-like) was decreased (median 0.1% versus 0.3%, *p* = 0.02) as was basophil infiltration (median 0.1% versus 0.5%, *p* = 0.002).

### The iTME of patients with MGUS and treated patients has similarities to that of healthy young donors

**Fig. 5:**
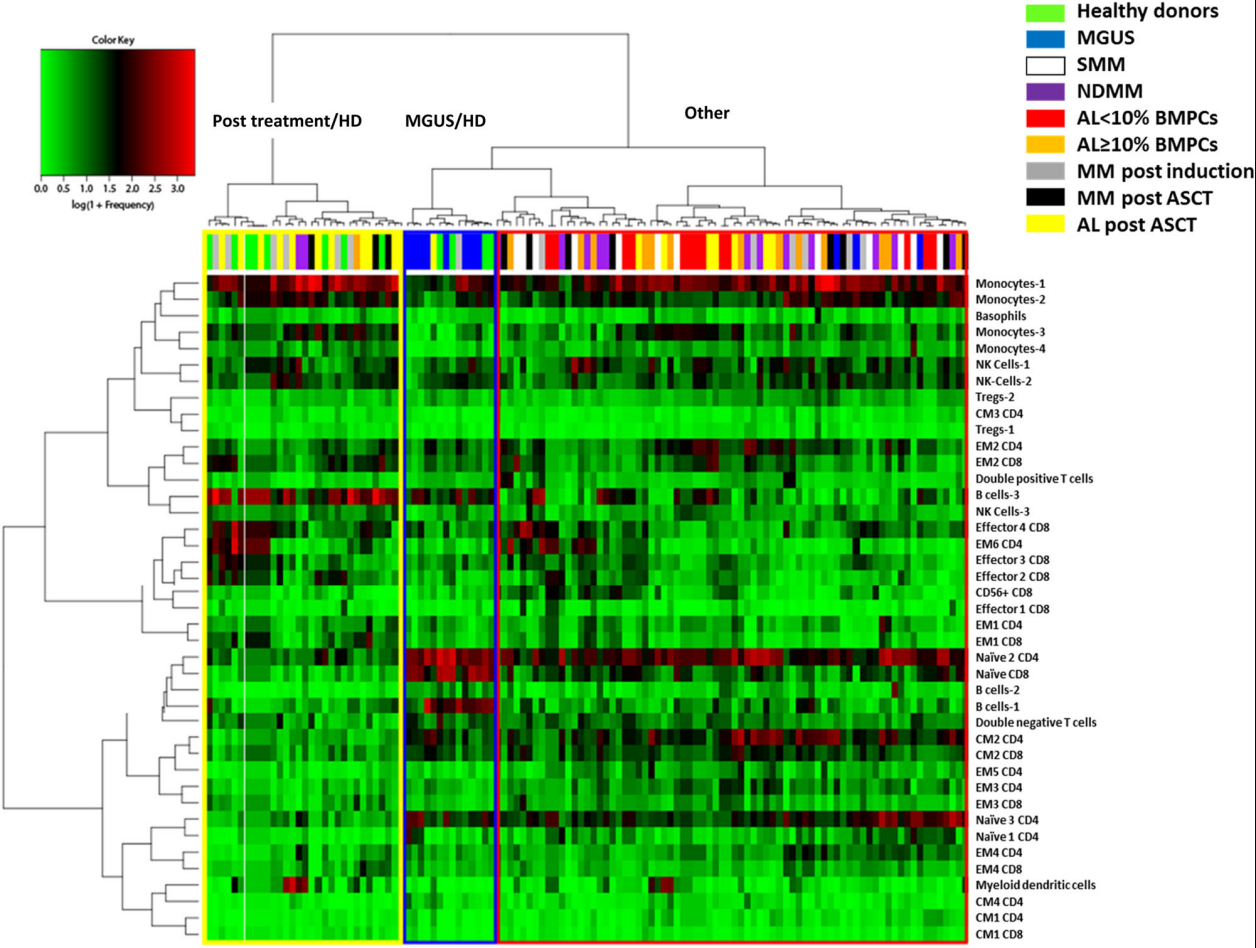
Hierarchical clustering of normalized immune subset frequencies (% of total CD45+ cells). AL newly diagnosed light chain amyloidosis, ASCT autologous stem cell transplant, BMPCs bone marrow plasma cells, CM central memory, EM effector memory, NDMM newly diagnosed multiple myeloma, NK natural killer, SMM smoldering multiple myeloma

**Fig. 6:**
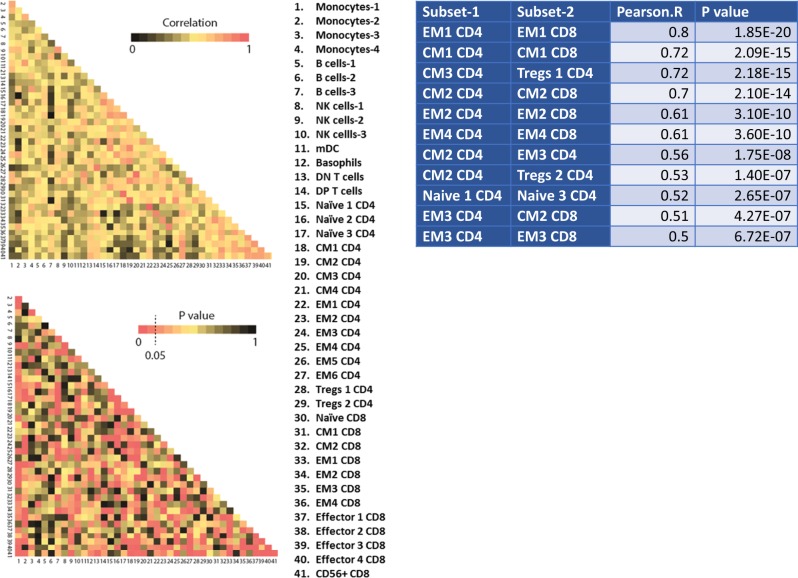
Relationships between immune subsets in the tumor microenvironment of dysproteinemias. Only significant correlations with a Pearson’s *R* > 0.5 are shown in the table. CM central memory, DN double negative, DP double positive, EM effector memory, mDC myeloid dendritic cells, NK natural killer

To identify patterns in the immune profiles of patients, we performed hierarchical clustering on a normalized frequency table (% of total CD45+ cells) of immune subsets of all samples. Two major patterns were evident (Fig. 5): patients with MGUS clustered with some HD samples and were predominantly characterized by low monocyte-1, monocyte-3 levels and high B-cell-1, B-cell-3, naïve 2 CD4, and naïve CD8 levels. Several post therapy patients (post-induction/ASCT) also clustered with some HD samples and were predominantly characterized by high B-cell-3 levels. This suggests that HD, MGUS patients, and post therapy patients represent distinct, and to some extent similar, immune states. This analysis also revealed potential correlations between immune subsets that were explored further using a correlation matrix (Fig. 6).

### Subsets with similar phenotypes tend to co-exist in the iTME of newly diagnosed patients

Several robust correlations were identified in the iTME of patients with newly diagnosed dysproteinemias. We noted that subsets with common phenotypes demonstrated strong correlations (CM1 CD4/CD8, CM2 CD4/CD8, EM2 CD4/CD8, EM4 CD4/CD8, Naïve 1, 3 CD4). This suggests that CD4 and CD8 T cells are exposed to similar regulatory networks in the iTME, which lead to similar polarization patterns. We noted that the terminally differentiated EM1 CD8 subset correlated with the regulatory^34^ CCR4+ EM1 CD4 subset. Finally, the activated CM2 CD4+ subset correlated with the Tregs2 and the terminally differentiated EM3 CD4 subsets. EM3 CD4 also showed robust correlations with the activated CM2 CD8 and EM3 CD8 subsets.

### High levels of terminally differentiated T cells and low levels of CD38/PD-1+ T cells are associated with shorter time to hematologic progression

**Fig. 7:**
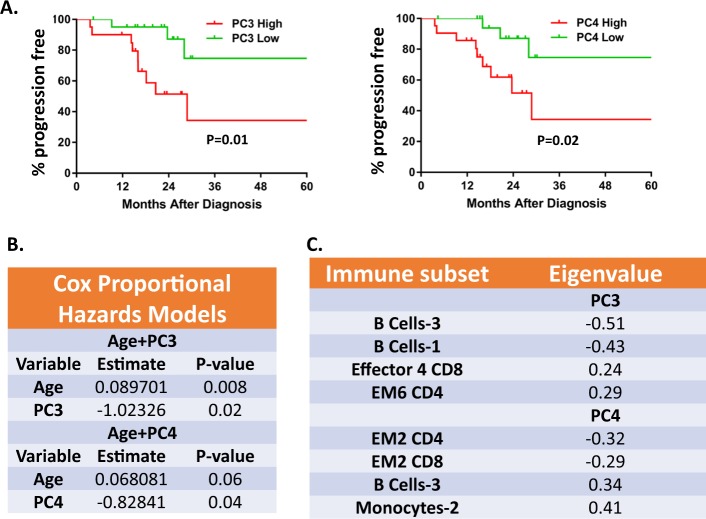
Correlation between the immune tumor microenvironment and time to hematologic progression. **a** Kaplan–Meier analysis of patients according to principal component (PC)-3 and PC-4 values. **b** Cox proportional hazards models testing the association of each PC with time to progression when adjusting for age. **c** Top contributing variables in PC3 and PC4 (absolute eigenvalue > 0.2)

In an effort to identify whether major drivers of immunomic variability are associated with time to hematologic progression we performed a PCA analysis in newly diagnosed patients with MM and AL amyloidosis. We considered MM and AL amyloidosis together for these analyses since time to hematologic progression should be unaffected by the different natural history of the latter due to organ infiltration by amyloid. No patients in either group died prior to hematologic progression. Furthermore, all patients were uniformly treated with early, upfront ASCT. A Cox proportional hazards model was utilized to test the association of each of the top five principal components (PCs) with time to hematologic progression (TTP) when adjusting for age as a confounding factor (Fig. 7).

Only PC3 and PC4 were significantly (negatively) associated with TTP. Next, we computed separate scores for each patient using the PC3 and PC4 models. Median cohort score for each model was assessed and patients were separated into PC-high and PC-low groups if their score was above or below the median score, respectively. High PC3 or PC4 scores had worse TTP. Cell subsets with the highest negative influence in TTP for PC3 included the terminally differentiated Effector 4 CD8 and EM6 CD4 and for PC4 the transitional B-Cell-3 and monocyte-2 subsets. Cell subsets with the highest positive influence in TTP for PC3 included B-cell 3 and B-cell 1 subsets and for PC4 the CD38/PD-1 double-positive EM2 CD4/CD8 subsets.

## Discussion

Using a large cohort of dysproteinemia patients analyzed with CyTOF, we have demonstrated the complexity of the T cell compartment in the iTME of patients with common dysproteinemias. We characterized novel phenotypes, some of which are unique to the iTME, with potential roles in tumor immunosurveillance and tumor immune escape. We found that AL amyloidosis has a distinct iTME compared to other dysproteinemias. We have shown that T cell immune senescence might be implicated in disease pathogenesis in patients with trisomies and MM progression after therapy. Finally, we demonstrate that the early post-ASCT period is associated with significant immunosuppression, which might have implications for the rational selection of post-ASCT therapies.

We were intrigued to note CCR5 expression in several effector T cell subsets. CCR5 ligands (CCL3 and CCL4) are produced by malignant plasma cells in the iTME of MM and are thought to be implicated in osteoclastogenesis^35,36^. CCR5 has conflicting roles in tumor immunosurveillance. CCR5 expression on malignant cells^37^ or immunosuppressive myeloid cells of the iTME^38^ promotes tumor escape from immune surveillance. However, CCR5 expression on CD4 T Cells is essential for activation of antigen-presenting cells and the resulting enhanced CD8 T cell cross-priming and tumor rejection in mouse models of malignancies^21^. This is thought to be mediated by the expression of CCR5 ligands CCL3 and CCL4 (also known as MIP-1alpha and MIP-1beta)^21^. Infiltration by CCR5+ lymphocytes is common in the microenvironment of tumors^39,40^ and has been associated with favorable prognosis^40^. We noted features of T cell exhaustion in some CCR5 expressing T cell subsets, which suggests that these populations of tumor-infiltrating lymphocytes might not be able to control tumor growth because they are exhausted.

Novel phenotypes included the CD38/PD-1+ EM2 and the innate like CM1 CD4 and CD8 T cell subsets. PD-1+ lymphocytes contribute the bulk of tumor-specific lymphocytes and are functionally impaired^41^. CD38/PD-1+ lymphocytes have only recently been identified in cancer^42^ and reactively increase after anti-PD-1 therapy^43,44^. Tumor escape from PD-1/PD-L1 blockade is thought to be mediated by CD38 expression on T cells^44^. These observations along with the fact that, in our study these subsets were not associated with worse TTP, might explain the lack of efficacy of PD-1 inhibitors as single-agents in this disease. However, more recently, combination of PD-1 inhibition with anti-CD38 monoclonal antibodies has been attempted in MM (NCT03023423) with encouraging early results. Eradication of these subsets rather than inhibition of PD-1 could explain the efficacy of this combination.

The two CM1 subsets characterized by HLA-DR/CD16 positivity are thought to represent “innate-like” T cell subsets^19,20^ that can be activated in the absence of T cell receptor stimulation^45^. These cells produce high levels of IFN-gamma and TNF-alpha upon stimulation through CD16^46^ and have a role in tumor immunosurveillance^19^. The CD8 subset was not detectable in the PB, suggesting that these cells are expanded only in the iTME. Both subsets were increased in the iTME of AL amyloidosis as were M2 polarized monocytes, CCR5+ and activated subsets. Amyloid clearance in tissues is thought to be mediated by phagocytosis of complement-opsonized amyloid deposits^47^ and increased macrophage phagocytosis of amyloid in tissues is a well-documented mechanism of amyloid clearance^30^. CD16 can be activated by complement-opsonized particles and could explain this observation.

Our results can help inform how anti-CD38 monoclonal antibodies can reshape the iTME if used at earlier stages of MM or AL amyloidosis. In relapsed MM for instance, anti-CD38 therapy depletes several immune suppressive subsets including regulatory B cells, T cells and myeloid-derived suppressor cells^48^. The same is likely to also be true at diagnosis and early after ASCT, where several myeloma-promoting subsets expressed high levels of CD38. CD38+ Tregs were increased early after ASCT and therefore incorporating anti-CD38 therapies in the maintenance setting is an attractive immunomodulatory option^49^. However, CD38 expression was absent in the IL-10 producing, CD25+ B regulatory subset and abundant in NK cell subsets and naïve B cell subsets that increased early after ASCT as part of normal immune reconstitution. The later suggests that anti-CD38 therapy early post-ASCT could increase infectious complications and diminish responses to vaccines^50^.

Senescent and exhausted T cell subsets were increased in patients with trisomies. The presence of trisomies is a negative prognostic factor in myeloma precursors but a positive one in NDMM, after therapy has been instituted. We have shown that patients with trisomies are especially sensitive to lenalidomide-based therapy^51^ and immunomodulation of these inactive subsets by lenalidomide might explain this^4,52^. Senescent/terminally differentiated T cell subsets were also found to be negatively associated with TTP. These cells are increased in MGUS and MM compared to age-matched healthy donors^53^, are not exhausted (PD-1−) and many of these subsets represent CD57+ clonal T cells^54^. It remains unclear what drives ineffective effector T cell expansion in this disease or how to prevent or reverse this process.

First line therapies, and especially ASCT, promoted several immune suppressive populations in the iTME. Lenalidomide and bortezomib-based induction increased M2-polarized monocytes and the senescent CM3 CD4 population but also the activated EM3 CD8 population. M2 macrophages are classically thought to promote MM cell growth and are associated with worse outcomes in MM^55^. However, lenalidomide can alter their function towards a more immunogenic phenotype^56^. The decrease in NK cell subsets was likely attributable to bortezomib^57^, since lenalidomide increases CD16+ and CD16− NK cells^4,57^. Early after ASCT several immunosuppressive subsets increased in the iTME, similar to what has been shown in the PB^58^. It should be noted that not all T-regulatory subsets are increased uniformly post-ASCT but only the highly immunosuppressive CD38/ICOS+ subset. Similar immunosuppressive T-regulatory subsets increase with lenalidomide maintenance post-ASCT, suggesting that perhaps a combination with anti-CD38 antibodies might be more effective.

Our study had several limitations. We did not include age-matched HD, so we could not identify which of these subsets at diagnosis were disease specific. However, the observation that several MGUS or treated patients clustered with young HD is novel and notable. We used cryopreserved samples and therefore myeloid populations could not be adequately assessed. For this reason we did not include markers for several myeloid populations of interest including myeloid-derived suppressor cells that are poorly preserved with cryopreservation^59^. Our panel did not include macrophage-specific markers and these cells were likely included in the CD163+ monocyte clusters and were not dissected further. However, T cell loss after cryopreservation is thought to be stochastic and most surface markers are recovered well after a brief post thaw rest period similar to our protocol. Finally, in our PB samples SMM and NDMM were overrepresented which could confound the results.

In conclusion, we have characterized, on a single cell level the complexity of the T cell compartment in the iTME of common dysproteinemias at diagnosis and after standard of care first line therapies. Immune-based therapies are revolutionizing the treatment landscape of patients with dysproteinemias and have a profound effect not only on the malignant clone but also the iTME. Understanding the complexity of the iTME at various stages of the disease using novel, systems-based, single-cell approaches will allow for the rational design of immunotherapy clinical trials in the future.

## Supplementary information

Supplemental Figure legends

Supplemental methods

Supplemental tables

Supplemental figure 1

Supplemental figure 2

Supplemental figure 3

Supplemental figure 4

Supplemental figure 5

Supplemental figure 6

Supplemental figure 7

Supplemental figure 8
