# Supplementary material for: Mass cytometry dissects T cell heterogeneity in the immune tumor microenvironment of common dysproteinemias at diagnosis and after first line therapies
Source: Blood Cancer J. 2019 Aug 28;9(9):72. doi: 10.1038/s41408-019-0234-4 (PMC6713712; doi:10.1038/s41408-019-0234-4)
Supplement: Supplementary file 1 — Supplemental Figure legends [file 41408_2019_234_MOESM1_ESM.docx]

**Supplemental figure 1**. Bone marrow samples included in this study. Matched peripheral blood sample numbers are included in parentheses. ASCT: autologous stem cell transplant, AL: Light chain amyloidosis, BMPCs: bone marrow plasma cells, MGUS: monoclonal gammopathy of undetermined significance, MM: multiple myeloma, SMM: smoldering multiple myeloma.

**Supplemental figure 2**. Schematic representation of the mass cytometry panel used and major phenotype key.

**Supplemental figure 3.** Gating strategy to identify CD45+, live single cells. Cell events were identified based on the lack of 140Ce expression (normalization bead marker). Single cell events were identified using all the linear Gaussian parameters (residual, center, offset, width, event length) and 191Ir expression as suggested by Fluidigm. Live cells were then selected based on the lack of cisplatin (195Pt) uptake.

**Supplemental figure 4.** Contour plots for major lineage markers of main cell subsets identified within the CD45+ component that underline the purity of the identified cell subsets. **mDC**: myeloid dendritic cells, **NK**: natural killer, **mPC**: malignant plasma cells.

**Supplemental figure 5.**The main immune components of the peripheral blood in common dysproteinemias. (A) viSNE map of 84,000 cells (2,000 cells/sample) from 35 dysproteinemias samples at various stages (newly diagnosed and post therapy) and 7 healthy donors. Lineage negative populations are not shown for clarity. (B) Heatmap showing expression of markers for each cluster; **mDC**: myeloid dendritic cells, **pDC**: plasmacytoid dendritic cells, **NK**: Natural killer cells,

**Supplemental figure 6.**CyTOF analyses of T Cells in the peripheral blood of common dysproteinemias. (A) and (C) viSNE map of 84,000 (2000 cells/ sample) CD8+ (A) and CD4+ (C) T cells from 35 dysproteinemias samples at various stages (newly diagnosed and post therapy) and 7 healthy donors. (B) and (D) Heatmap of CD8+ (B) and CD4+ (D) T cells, respectively, showing expression of markers for each cluster. Markers not expressed on T cells are not shown for clarity. **CM**: central memory, **EM**: Effector memory, **Tregs**: T regulatory cells

**Supplemental figure 7.** Immune subsets with extreme low/high abundance for one patient with high risk smoldering myeloma demonstrating spontaneous hematologic response (“remission”) over time are shown. Immunosuppressive subsets such as M2 polarized monocytes and terminally differentiated (EM6 CD4, EM1 CD8, effector 1 CD8, effector 2 CD8, effector 3 CD8, CD56+ CD8) T Cell subsets have the lowest or near lowest abundance in the group, whereas immune surveilling/activated subsets (CM1 CD4, CM2 CD4) have the highest or near highest abundance. **CM**: central memory, **EM**: effector memory, **NK**: Natural killer

**Supplemental figure 8.** Boxplots showing differential abundance of immune subsets after lenalidomide-based induction chemotherapy in patients with newly diagnosed multiple myeloma. T cell subset frequencies are shown as % of total CD3 cells. **CM**: central memory, **EM**: effector memory, **MM**: multiple myeloma, **NDMM**: newly diagnosed multiple myeloma, **NK**: Natural killer.
