# Supplementary material for: Mass cytometry dissects T cell heterogeneity in the immune tumor microenvironment of common dysproteinemias at diagnosis and after first line therapies
Source: Blood Cancer J. 2019 Aug 28;9(9):72. doi: 10.1038/s41408-019-0234-4 (PMC6713712; doi:10.1038/s41408-019-0234-4)
Supplement: Supplementary file 2 — Supplemental methods [file 41408_2019_234_MOESM2_ESM.docx]

*Antibody and sample preparation*

For primary conjugations, purified antibodies were obtained in carrier protein-free PBS and labeled using the X8 polymer MaxPAR antibody conjugation kit (Fluidigm) according to the manufacturer’s protocol. The antibody panel was designed using the web-based Fluidigm panel designer to select channels with optimal signal and minimal background from oxidation, isotopic impurity or abundance sensitivity. All antibodies were titrated to optimal staining concentrations using primary human bone marrows of patients with MM. Antibody master mixes were prepared fresh for each experiment.

All BM and PB samples were processed identically. Mononuclear cells were obtained after ACK lysis of BM and PB samples and were viably frozen in RPMI 1640, 20%FBS, 10% DMSO. Cryopreserved cells were resuscitated for mass cytometry analyses by rapid thawing and were rested in RPMI 1640 (20% FBS) for 60 minutes prior to staining. Staining was performed using Fluidigm’s protocol. Briefly, 1-3 million cells were stained for viability with 5mM cisplatin for 5 mins at room temperature and quenched with cell staining medium (CSM, Fluidigm). Cells were then incubated for 10 mins at room temperature with human FcR blocking reagent (Biolegend) and then stained with the surface antibody cocktail for 60mins at 4^o^C with gentle agitation. Finally, cells were washed twice with CSM, fixed with 1.6% PFA, washed with CSM and resuspended in 1:1000 solution of Iridium intercalator diluted in MaxPar Fix and Perm buffer (Fluidigm) for 20mins at room temperature. Prior to acquisition, cells were washed twice in CSM and twice in deionized water and were then diluted to a concentration 0.5million cells/ml in water containing 10% of EQ 4 Element Beads (Fluidigm). Cells were filtered through a 35μm membrane prior to mass cytometry acquisition. Samples were then acquired on a Helios mass cytometer.
