# Supplementary material for: Mass cytometry dissects T cell heterogeneity in the immune tumor microenvironment of common dysproteinemias at diagnosis and after first line therapies
Source: Blood Cancer J. 2019 Aug 28;9(9):72. doi: 10.1038/s41408-019-0234-4 (PMC6713712; doi:10.1038/s41408-019-0234-4)
Supplement: Supplementary file 3 — Supplemental tables [file 41408_2019_234_MOESM3_ESM.docx]

**Supplemental table 1**.CytOF antibody panel

| **Target** | **Clone** | **Metal tag** | **Source** |
| --- | --- | --- | --- |
| CD45 | HI30 | 89Y | Fluidigm |
| HLA-DR | Tu36 | 114Cd | Thermo Fisher |
| CD196 (CCR6) | G034E3 | 141Pr | Fluidigm |
| CD11a | HI111 | 142Pr | Fluidigm |
| CD195 (CCR5) | NP-6G4 | 144Nd | Fluidigm |
| CD4 | RPA-T4 | 145Nd | Fluidigm |
| CD278 (ICOS) | C398.4A | 148Nd | Fluidigm |
| CD25 (IL-2R) | 2A3 | 149Sm | Fluidigm |
| CD14 | M5E2 | 151Eu | Fluidigm |
| CD57 | HCD57 | 152Eu | Biolegend* |
| CD45RA | HI100 | 153Eu | Fluidigm |
| CD163 | GH1/61 | 154Sm | Fluidigm |
| CD279 (PD-1) | EH12.2H7 | 155Gd | Fluidigm |
| CD183 (CXCR3) | G025H7 | 156Gd | Fluidigm |
| CD194 (CCR4) | 205410 | 158Gd | Fluidigm |
| CD197 (CCR7) | G043H7 | 159Tb | Fluidigm |
| CD28 | CD28.2 | 160Gd | Fluidigm |
| CD152 (CTLA4) | 14D3 | 161Dy | Fluidigm |
| CD11c | Bu15 | 162Dy | Fluidigm |
| CD56 | NCAM16.2 | 163Dy | Fluidigm |
| CD45RO | UCHL1 | 165Ho | Fluidigm |
| CD44 | BJ18 | 166Er | Fluidigm |
| CD27 | O323 | 167Er | Fluidigm |
| CD138 | DL-101 | 168Er | Fluidigm |
| CD19 | HIB19 | 169Tm | Fluidigm |
| CD3 | UCHT1 | 170Er | Fluidigm |
| CD38 | HIT2 | 172Yb | Fluidigm |
| CD8a | RPA-T8 | 174Yb | Biolegend* |
| CD274 (PD-L1) | 29E.2A3 | 175Lu | Fluidigm |
| CD127 (IL-7R) | A019D5 | 176Yb | Fluidigm |
| CD16 | 3G8 | 209Bi | Fluidigm |

* These antibodies were conjugated to their respective metal tags using the X8 polymer MaxPAR antibody conjugation kit (Fluidigm) according to the manufacturer’s protocol.

**Supplemental table 2**. Frequencies of CD45+ and CD3+ immune clusters identified in the marrow immune microenvironment.

| **Cluster type** | **% Median frequency (range)** | | | | |
| --- | --- | --- | --- | --- | --- |
|  | **Healthy donors** | **MGUS** | **SMM** | **Newly diagnosed Myeloma** | **Newly diagnosed AL amyloidosis** |
| **CD45+** | **of total CD45+ cells** | | | | |
| Monocytes-1 | 3 (1.4-14) | 8.7 (1.8-22.8) | 6 (1.6-12.4) | 8.7 (1-28.4) | 8.7 (3.5-27) |
| Monocytes-2 | 2.3 (0.4-6.3) | 4.6 (2.2-9.8) | 4.9 (2.4-6.7) | 4.8 (0.5-11.1) | 3.1 (1-9.9) |
| Monocytes-3 | 0.1 (0-3.1) | 0.8 (0.1-7.5) | 0.9 (0-5.3) | 1.1 (0.1-4.2) | 2.5 (0.8-76) |
| Monocytes-4 | 0.1 (0-1.3) | 0.5 (0.2-1.4) | 0.6 (0.1-8.5) | 0.5 (0.1-1.9) | 0.8 (0.3-2.1) |
| B-Cells-1 | 6.9 (1-20) | 0.6 (0.1-2.5) | 0.9 (0.1-3) | 1.3 (0.3-6.2) | 1 (0.1-8) |
| B-Cells-2 | 1 (0.3-3.4) | 0.5 (0-9.8) | 0.3 (0.1-1.9) | 0.5 (0-2) | 0.5 (0-2.1) |
| B-Cells-3 | 3.7 (1.9-8.4) | 0.5 (0.1-10.6) | 1.4 (0.1-4.6) | 0.6 (0.1-5.1) | 1.3 (0-13) |
| NK-Cells-1 | 1.6 (0.2-6.9) | 3.2 (1.2-10.5) | 3.2 (1.4-13.5) | 2.9 (0.3-8.1) | 2.4 (0.9-7.4) |
| NK Cells-2 | 2.4 (0.2-4.3) | 2.4 (0.8-7.3) | 2.8 (0.8-6) | 2.8 (0.5-5) | 2.4 (1.2-4.1) |
| NK-Cells-3 | 0.6 (0-1.9) | 0.4 (0.3-2.1) | 0.6 (0.3-1.9) | 1 (0.2-4.8) | 0.7 (0.2-2.9) |
| mDC | 0 (0-0.3) | 0.2 (0-8.1) | 0.1 (0-5.6 | 0.1 (0-10.5) | 0.2 (0-10%) |
| Basophils | 0.5 (0-0.7) | 0.3 (0.1-1.2) | 0.3 (0-0.9) | 0.2 (0-0.7) | 0.5 (0-2.3) |
| T Cells | 57.1 (45.6-70) | 45.7 (175-62.2) | 50 (39.1-73.9) | 45 (17.4-70.8) | 51.8 (26.1-71..1 |
| DP T Cells | 0.6 (0.3-0.8) | 0.4 (0.1-0.6) | 0.5 (0.1-3.1) | 0.4 (0.1-10.2) | 0.7 (0.1-5.6) |
| DN T Cells | 12 (0.8-7.7) | 1.3 (0.4-3.1) | 1.5 (0.6-3.4) | 1.1 (0.6-3.4) | 1.3 (0.4-3.9) |
| **CD8+** | **of total CD3+ cells** | | | | |
| Naïve | 22 (7.4-33) | 3.1 (0.3-8.3) | 4.4 (0.7-9) | 4.5 (0.8-13) | 4.5 (0.8-11) |
| CM1 | 0.1 (0-0.9) | 0.2 (0.1-0.5) | 0.4 (0-1) | 0.3 (0-0.5) | 0.5 (0.1-2.4) |
| CM2 | 4 (2.1-7.1) | 5.5 (1.5-13.2) | 5.5 (3.1-9.3) | 4.8 (1.5-8.6) | 5.2 (1.6-13.5) |
| EM1 | 1 (0.1-2.5) | 1 (0.1-5.9) | 1.3 (0.3-12.5) | 0.6 (0.1-4.9) | 1.3 (0.2-12.4) |
| EM2 | 1.9 (0.7-4.2) | 2.1 (0.3-3) | 2.5 (0.4-6.4) | 3.4 (0.5-10.2) | 2.7 (0.8-16.2) |
| EM3 | 1.3 (0.7-2.7) | 2 (0.2-5.9) | 3.1 (0.6-9.3) | 2.2 (0.8-3.3) | 2.4 (1.16-14.8) |
| EM4 | 0.9 (0.2-2.7) | 1.7 (0.4-5.9) | 2.5 (0.4-3.9) | 1.4 (0.5-7.8) | 1.4 (0.3-4.2) |
| Effector 1 | 0.1 (0-1.7) | 0.3 (0-3.2) | 0.3 (0-5) | 0.3 (0-0.8) | 0.3 (0.1-4.3) |
| Effector 2 | 0.8 (0.4-3.4) | 0.9 (0.2-7.3) | 1.9 (0-5.6) | 0.9 (0.1-3.6) | 1.7 (0.1-8.8) |
| Effector 3 | 2.3 (1-6.4) | 1.9 (0.1-7.7) | 1.6 (0.3-6.6) | 2.2 (0.3-5.4) | 1.9 (0.5-5.9) |
| Effector 4 | 2.9 (0.6-6.4) | 3.5 (0.6-6.9) | 4.7 (1.1-22.6) | 4.4 (1-20.6) | 2.4 (0.6-8.1) |
| CD56+ | 0.7 (0-1.8) | 1.5 (0-4.3) | 1.6 (0.3-6.3) | 2.3 (0.1-10.7) | 0.9 (0.1-7.3) |
| **CD4+** | **Of total CD3+ cells** | | | | |
| Naïve 1 | 0.7 (0-11) | 1.6 (0.1-7.5) | 1.3 (0.2-11.3) | 1.8 (0.3-5.3) | 1.6 (0.1-5.4) |
| Naïve 2 | 20 (13-32) | 13.2 (7.3-26.6) | 15.9 (2.9-25.3) | 14 (2.7-26) | 15.6 (2.6-30) |
| Naïve 3 | 7.8 (2-18.5) | 12.1 (1.8-29.7) | 10.2 (2.6-32.7) | 9.3 (4.1-21.2) | 6.4 (0.8-24.2) |
| CM1 | 0.1 (0-1.3) | 0.3 (0.1-0.8) | 0.4 (0.1-1.3) | 0.4 (0-1) | 1 (0.1-5.7) |
| CM2 | 5.2 (1.9-11) | 12.1 (3.8-27.2) | 8 (2.5-24.1) | 6.6 (3-31) | 9.3 (1.9-30) |
| CM3 | 0.1 (0-0.6) | 0.6 (0.1-1.2) | 0.4 (0.2-0.8) | 0.5 (0.1-1.7) | 0.4 (0.1-1) |
| CM4 | 0.6 (0.1-2.9) | 0.3 (0.1-2.4) | 0.3 (0-1.1) | 0.5 (0.2-2.9) | 0.5 (0.1-4.4) |
| EM1 | 1.2 (0.2-2.6) | 1.4 (0.6-5.3) | 1.8 (0.5-9.6) | 1 (0.1-5) | 2.8 (0.5-12.9) |
| EM2 | 2.1 (1-6.6) | 3.7 (1.1-9.4) | 3.6 (0.3-10.1) | 5.9 (0.9-17.4) | 4.7 (0.7-20.1) |
| EM3 | 1 (0.3-2.9) | 2.3 (0.5-7.9) | 2.3 (0.1-5.9) | 1.4 (0.2-4.9) | 2.9 (0.7-8.1) |
| EM4 | 1.2 (0.3-3.4) | 3.2 (1.2-25.7) | 2.6 (0.8-7.5) | 2.5 (0.8-7.6) | 2.2 (0.7-11.7) |
| EM5 | 0.5 (0.1-1.6) | 2.1 (0.4-6.3) | 1.3 (0.2-3.9) | 1 (0.1-3.5) | 2.2 (0.3-10.6) |
| EM6 | 0.4 (0.1-1.3) | 0.6 (0-7.1) | 1.7 (0-8.1) | 0.5 (0.2-13.2) | 0.5 (0.1-9.3) |
| Tregs 1 | 0.2 (0-0.4) | 0.3 (0-0.9) | 0.3 (0.1-1.1) | 0.4 (0.1-1.3) | 0.3 (0.1-1.1) |
| Tregs 2 | 1 (0.3-2.7) | 1.4 (0.5-3.8) | 2 (0.5-2.6) | 1.9 (0.5-7.1) | 1.6 (0.6-2.8) |

**CM**: central memory, **DP**: double positive, **DN**: double negative, **EM**: effector memory, **Tregs**: T regulatory cells.
