# Supplementary figures and images for: Mass cytometry dissects T cell heterogeneity in the immune tumor microenvironment of common dysproteinemias at diagnosis and after first line therapies
Source: Blood Cancer J. 2019 Aug 28;9(9):72. doi: 10.1038/s41408-019-0234-4 (PMC6713712; doi:10.1038/s41408-019-0234-4)

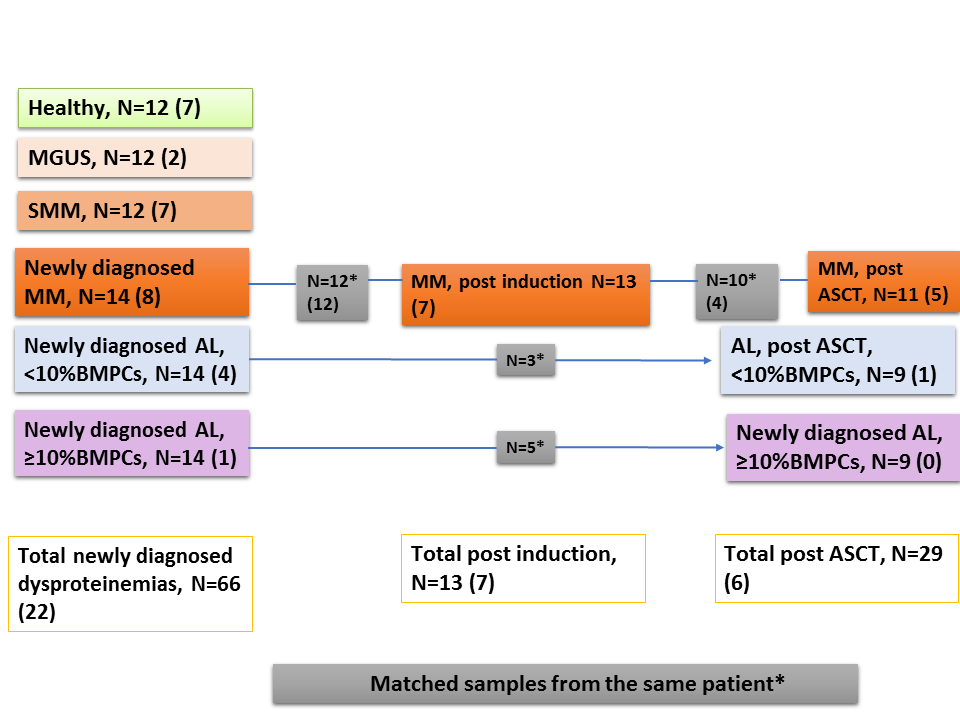

Supplement: Supplementary file 4 — Supplemental figure 1 [file 41408_2019_234_MOESM4_ESM.tif]

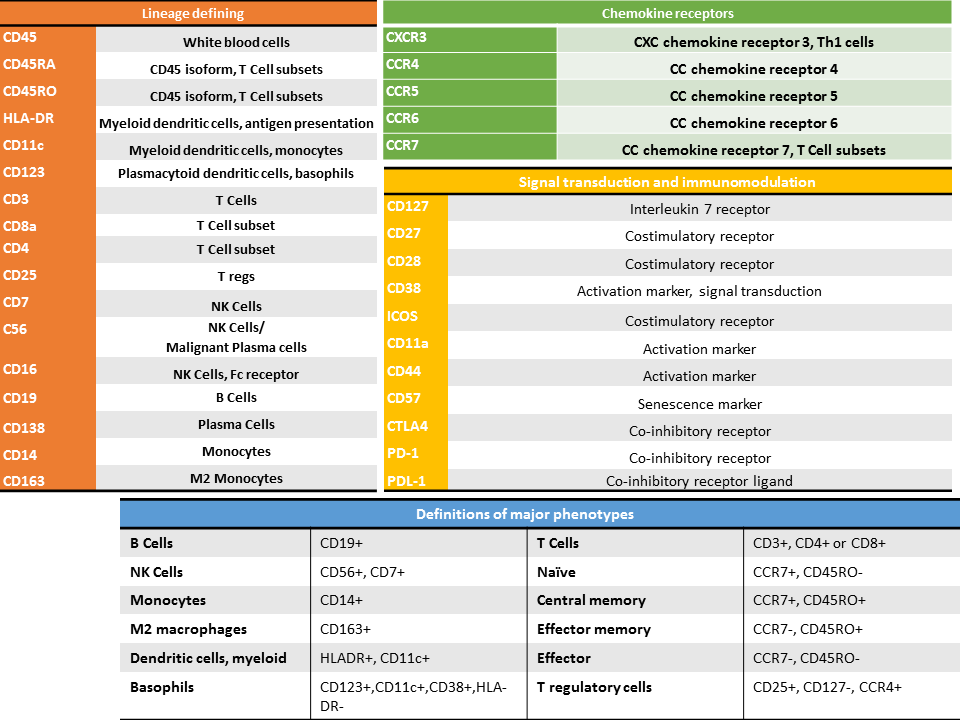

Supplement: Supplementary file 5 — Supplemental figure 2 [file 41408_2019_234_MOESM5_ESM.tif]

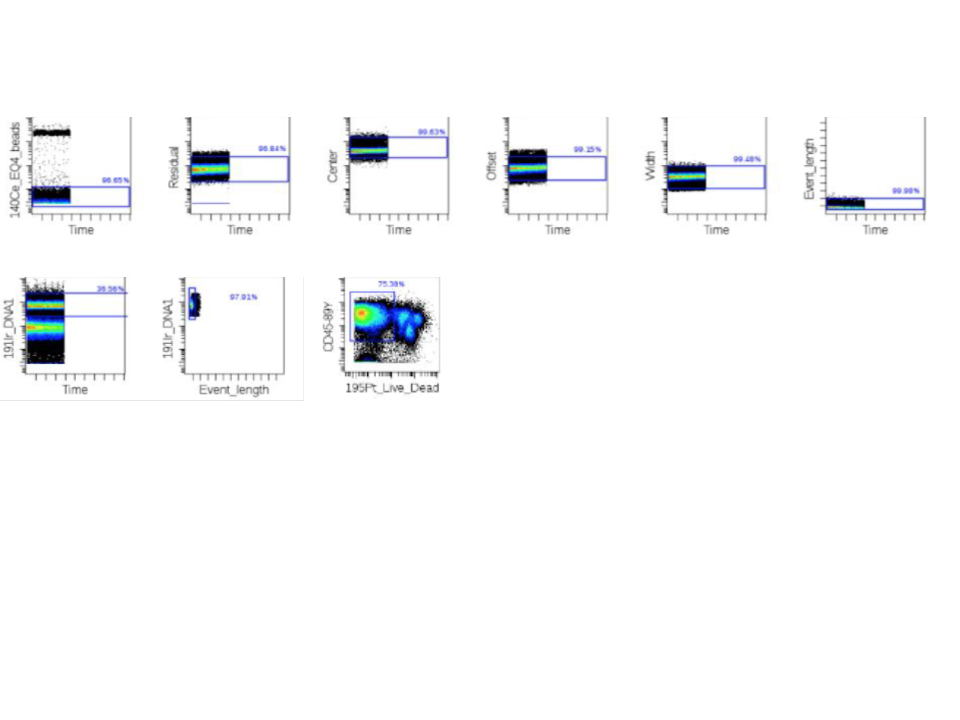

Supplement: Supplementary file 6 — Supplemental figure 3 [file 41408_2019_234_MOESM6_ESM.tif]

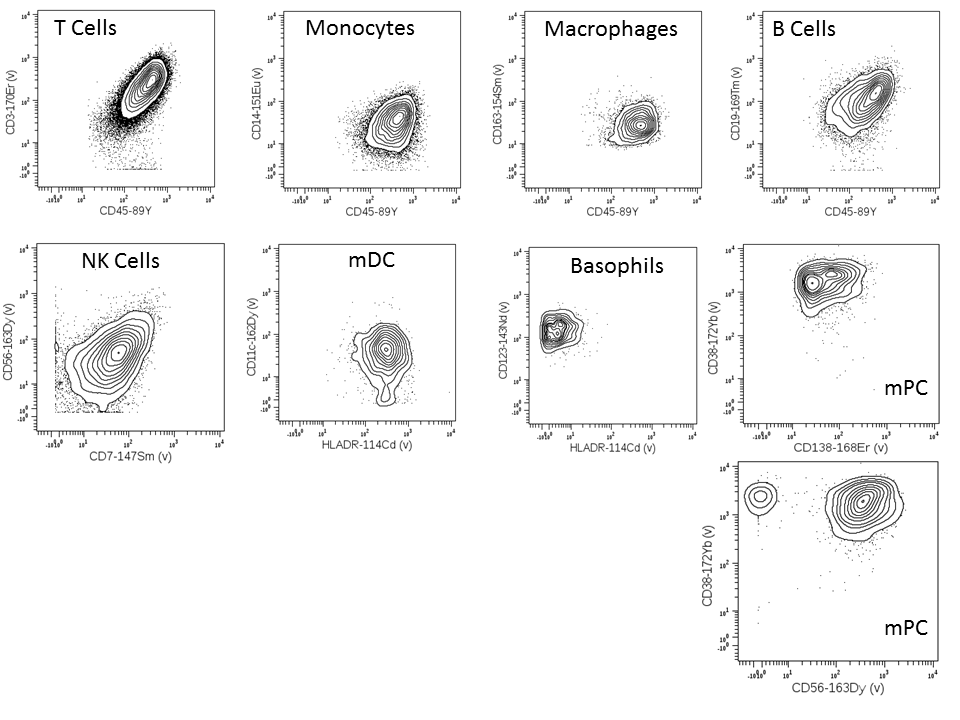

Supplement: Supplementary file 7 — Supplemental figure 4 [file 41408_2019_234_MOESM7_ESM.tif]

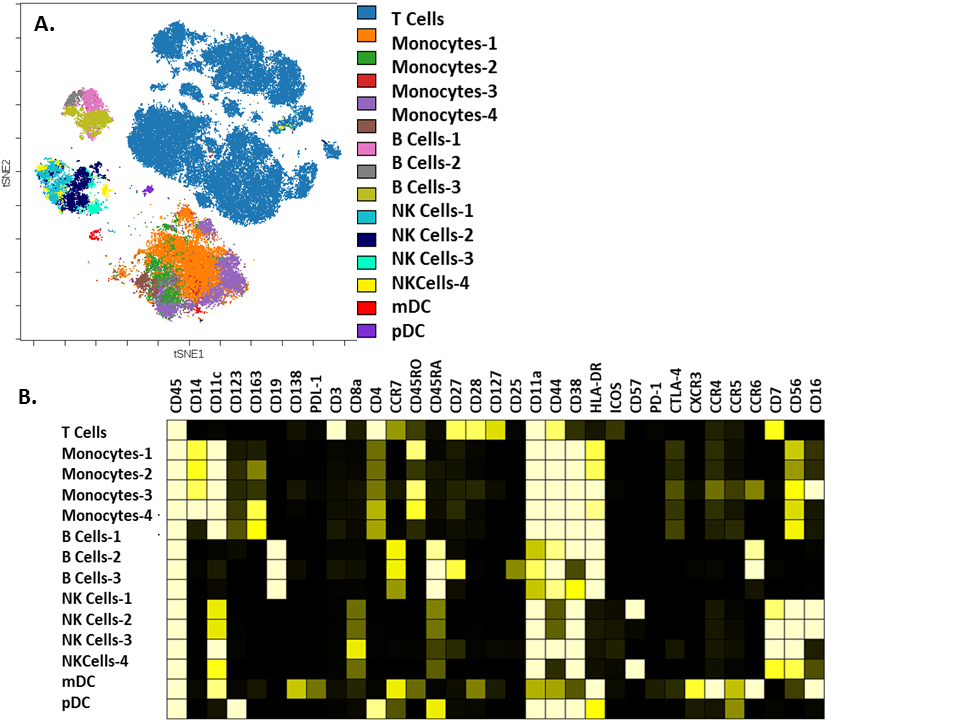

Supplement: Supplementary file 8 — Supplemental figure 5 [file 41408_2019_234_MOESM8_ESM.tif]

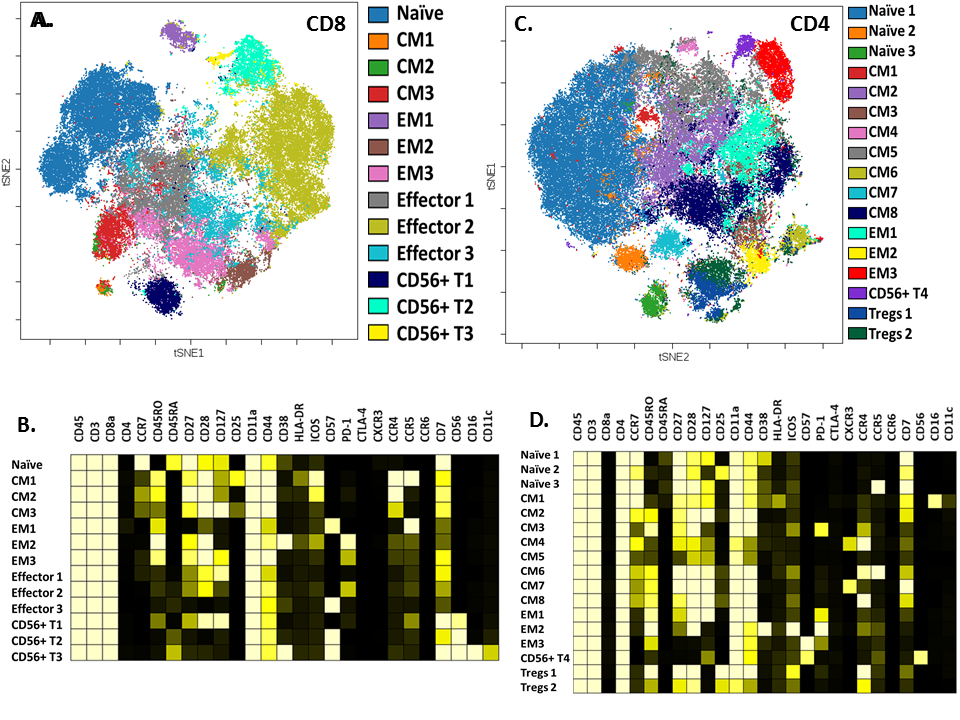

Supplement: Supplementary file 9 — Supplemental figure 6 [file 41408_2019_234_MOESM9_ESM.tif]

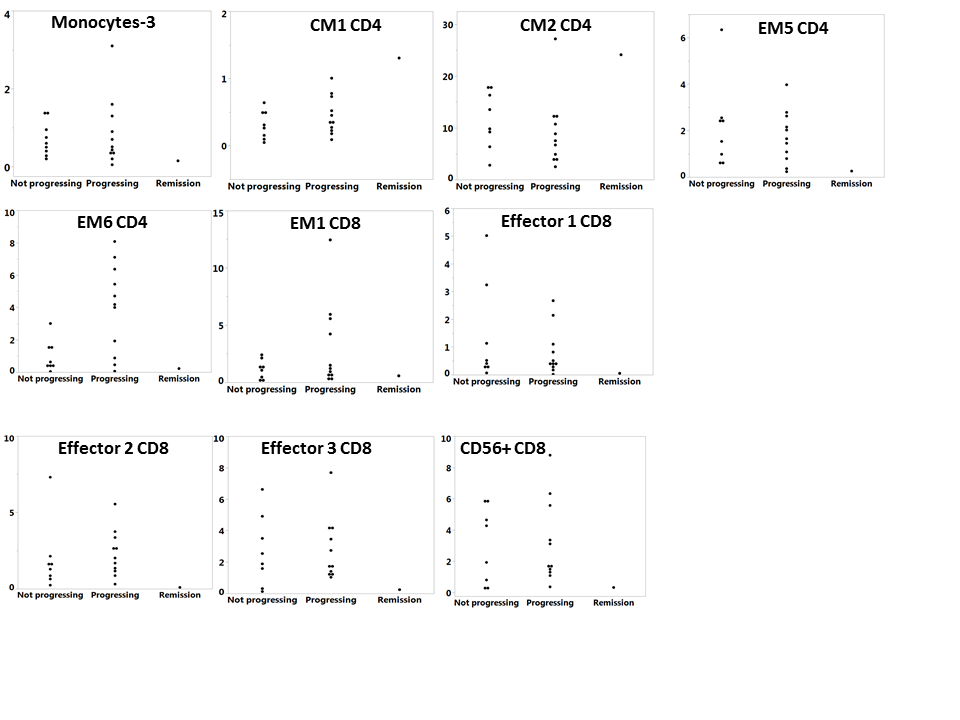

Supplement: Supplementary file 10 — Supplemental figure 7 [file 41408_2019_234_MOESM10_ESM.tif]

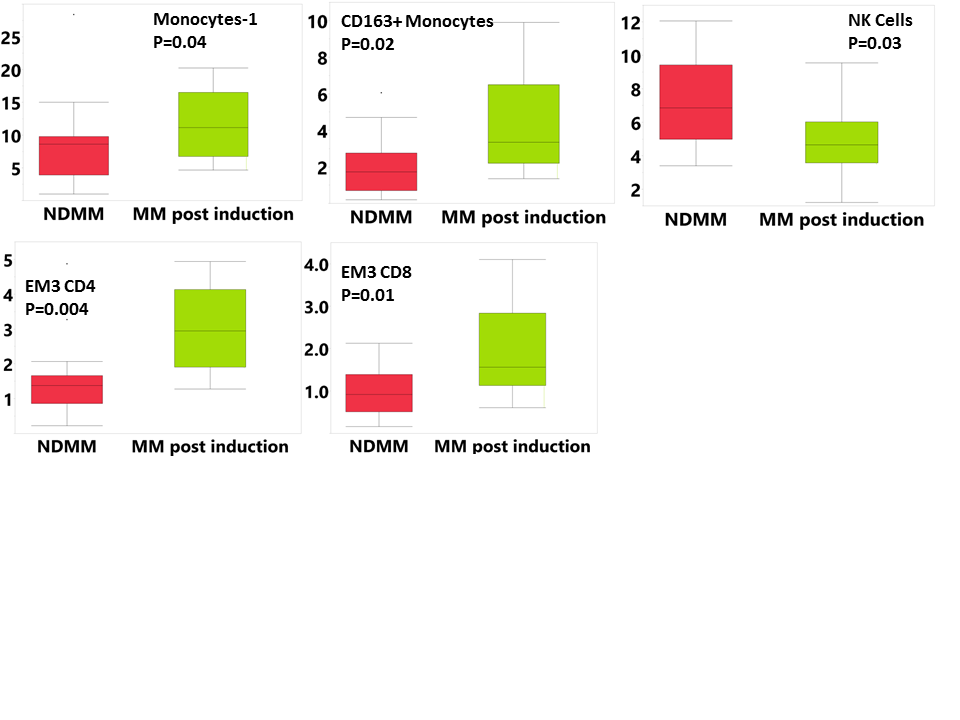

Supplement: Supplementary file 11 — Supplemental figure 8 [file 41408_2019_234_MOESM11_ESM.tif]
